# Retrospective Analysis of Central Nervous System Diseases in Dogs, with Special Focus on Non-Suppurative Encephalomyelitis (1962–2022)

**DOI:** 10.3390/vetsci12090869

**Published:** 2025-09-08

**Authors:** Inga Marie Nägler, Adnan Fayyad, Christina Puff, Wolfgang Baumgärtner, Peter Wohlsein

**Affiliations:** 1 Department of Pathology, University of Veterinary Medicine Hannover, 30559 Hannover, Germany; inga.naegler@tiho-hannover.de (I.M.N.); adnanf@najah.edu (A.F.); christina.puff@tiho-hannover.de (C.P.); peter.wohlsein@tiho-hannover.de (P.W.); 2 Center for Systems Neuroscience, University of Veterinary Medicine Hannover, 30559 Hannover, Germany; 3 Department of Veterinary Medicine, Faculty of Agriculture and Veterinary Medicine, An-Najah National University, Nablus P405, Palestine

**Keywords:** canine distemper, dog, encephalomyelitis, neuropathology, pseudorabies, retrospective study, viral encephalitis

## Abstract

Studies spanning decades provide important information about the epidemiology and occurrence of a broad range of diseases that affect the central nervous system (CNS) of dogs. This study analyzed records and formalin-fixed paraffin-embedded (FFPE) CNS tissue samples from necropsied dogs with neuropathologic changes between 1962 and 2022. A total of 134,854 animals, including 20,117 dogs, were submitted for necropsy during this time span. Of these dogs, 2646 displayed alterations of the CNS. Degenerative and non-suppurative inflammatory lesions were the most common changes, accounting for 35.6% and 28.6%, respectively. Vascular diseases, neoplasms, congenital malformations, and suppurative inflammation represented 13.8%, 8.6%, 7.2%, and 5.4% of cases, respectively. Morbillivirus canis, the agent of canine distemper, was the most commonly diagnosed. The second most commonly detected virus, varicellovirus suidalpha1, the agent of pseudorabies, occurred almost exclusively between the mid-1970s and 1990s. Other pathogens, including Lyssavirus rabies, canine herpes virus, tick-borne encephalitis virus, apicomplexan parasites, such as *Neospora caninum* and *Toxoplasma gondii*, as well as fungal and other parasitic infections, were less frequently diagnosed. Interestingly, 47.6% of cases with non-suppurative inflammation remained etiologically undetermined. This study provides insights into the epidemiology of canine neurotropic infections and shows the value of FFPE material for investigations of past disease outbreaks.

## Simple Summary

Retrospective evaluations of medical cases can provide valuable information about the incidence of diseases in a population. In this study, archived files and necropsy findings of formalin-fixed, paraffin wax-embedded tissue specimens from 2646 dogs out of 20,117 necropsied dogs were found to be affected by a central nervous disorder over a period of 61 years from 1962 to 2022. In addition to various non-inflammatory diseases, 758 (28.6%) dogs were diagnosed with a non-suppurative inflammation of the central nervous system. In 397 (52.4%) of these cases, a specific etiology was identified, most commonly canine distemper and pseudorabies (also termed Aujeszky’s disease). Especially the pseudorabies cases cumulated in one distinct wave between the mid-1970s and 1990s, while canine distemper cases were found more evenly distributed with less clearly separated peaks, in the late 1960s and 1990s. This study shows the great value of long-term stored formalin-fixed, paraffin wax-embedded-tissue specimens for retrospective studies using different histologic stainings and immunohistochemistry for the detection of previously occurring disease outbreaks.

## 1. Introduction

The brain and spinal cord are affected by a wide range of diseases and different underlying pathogenetic mechanisms, resulting in diverse pathologic manifestations [1,2,3,4,5]. These include infectious and non-infectious inflammatory lesions, congenital malformations, neoplasms, and other poorly understood processes. To categorize this variety of different changes, veterinary clinical neurologists often rely on the VITAMIN D scheme, an acronym for vascular disorders, inflammatory changes, trauma, anomalies (congenital malformations), metabolic and toxic alterations, idiopathic diseases, neoplasms, and degeneration. Inflammation might be further divided into suppurative and non-suppurative types [6].

Non-suppurative inflammation of the central nervous system (CNS) in dogs continues to pose a diagnostic and therapeutic challenge, as it remains etiologically undetermined in many cases [7,8,9,10]. Besides well-known viruses—for which a precise diagnosis is crucial due to either their high contagiousness, as seen in canine distemper virus (CDV; new terminology Morbillivirus canis), or their zoonotic potential, e.g., rabies virus (Lyssavirus rabies)—previously unrecognized pathogens might also play a role in both individuals as well as in outbreaks in larger populations of animals [9,11,12,13,14]. Non-infectious, autoimmune disease patterns must also be considered as causes of non-suppurative encephalitis and myelitis [7,8,15,16,17,18,19].

To analyze the prevalence of these different patterns of neuropathologic diseases, large-scale retrospective studies spanning several decades can be of great benefit. Studies evaluating either clinical or pathological data over a long time span in dogs are often focused on a single breed or disease [20,21,22,23,24]. A study from Sweden, which associated age, sex, and breed with causes of morbidity and mortality in 1996, identified that diseases of the integumentary system were recognized as the most frequent reason for veterinary treatment, while neurologic diseases were less relevant [25]. A study from Hannover, Germany, based solely on the written documentation of pathologic findings, analyzed all dogs submitted for necropsy between 1938 and 1963. The investigation revealed that CDV was the most common cause of death in dogs and was suspected in 12% of all cases, with the nervous form of distemper being the most common manifestation, noted in 4.5% of all examined dogs [26]. Another study from Switzerland including clinical cases and dogs submitted for necropsy covered a 12-year period between 1989 and 2000 of canine neurologic cases and found degenerative changes to be the most frequent lesion [27]. An approach of categorizing neurologic cases in cats and dogs between 1998 and 2003 based on pathologic data, including both necropsied animals and biopsies, was performed in Hannover, Germany, focusing on non-suppurative encephalitis/myelitis cases and their possible infectious origin, with CDV being the most frequently diagnosed agent in dogs; however, most cases (75%) remained etiologically undetermined [28].

Other studies of neurologic canine cases, with a special focus on meningoencephalitis of unknown origin, relied entirely on clinical data [7,16]. A study from the USA covering neuro-inflammatory lesions in necropsied dogs between 2008 and 2019 found 111 cases to be idiopathic, and 96 cases were classified as having an infectious etiology. There were more bacterial than viral infections, and canine herpesvirus (CHV) was the most frequently diagnosed virus infection. The study defined any case without a diagnosed infectious agent as being of non-infectious origin [29].

The most commonly described virus causing non-suppurative encephalomyelitis in dogs is CDV, which may cause systemic disease, with the CNS being one of the possible affected organs [1,30]. Due to widespread vaccination, the disease has become rare in dogs in Germany [11,30]. Similarly, CHV causes a systemic disease, which might include the CNS. In dogs, it may affect neonates and may also cause abortions and stillbirths [31,32]. Rabies virus with its zoonotic potential is the most dangerous agent causing encephalitis in dogs. All mammalian species are susceptible to rabies, albeit with differences in susceptibility [14,33,34,35]. Due to extensive vaccinations of pets and foxes, Germany is free of the sylvatic form of rabies; however, various serotypes of European bat lyssavirus are still circulating in bats [36,37]. Other important viruses causing encephalomyelitis in dogs include Suid Herpesvirus 1 (SHV-1; new terminology Varicellovirus suidalpha1) [12], the causative agent of pseudorabies. This disease is primarily transmitted by infected pork and is clinically similar to rabies [38].

A comprehensive and inclusive retrospective investigation into the occurrence and variations in disease manifestations depends on the availability of archived pathologic reports, formalin-fixed, paraffin-embedded (FFPE) material and hematoxylin and eosin (HE)-stained slides. The objective of this study was to analyze neuropathologic cases in dogs between 1962 and 2022 and to categorize the morphologic changes. In cases with non-suppurative encephalomyelitis, the most common causes were evaluated and assessed regarding their temporal incidence.

## 2. Materials and Methods

### 2.1. Archived Documentation of Animals

The Department of Pathology, University of Veterinary Medicine Hannover, Hannover, Germany, has housed a systematic archive of FFPE material since 1962. Moreover, the recorded written documentation of necropsy cases, including anamnestic data and macroscopic and histologic findings, and results of additional investigations, including microbiological and parasitological findings, dates back to the 1930s. In the present study, the archived records and FFPE material of all dogs submitted for necropsy between 1962 and 2022 were screened for those animals exhibiting neuropathologic changes. The search criteria included gross and/or histologic lesions of the CNS. The anamnestic and clinical histories were not included in the case selection process due to great variability in the detailed recording, ranging from scant or completely lacking information in many cases.

Neuropathologic cases were further categorized according to a modified VITAMIN D scheme (Table 1) that allowed classification on the basis of macroscopic and histologic alterations without considering the medical history and clinical findings. A definition of each category is found in Table 1. Cases were categorized based on their most prominent finding as vascular disease, inflammatory disease, anomaly, neoplasia, or degenerative disease. Inflammatory diseases were further divided into suppurative and non-suppurative inflammation. Some cases originally diagnosed as encephalitis could not be further assessed regarding the type of inflammation due to insufficient documentation and unavailability of tissue sections and FFPE material. These cases are referred to as unclassified inflammation in the final analysis. In addition, neoplasms were divided into primary and secondary neoplasms without further phenotypical classification. Any anamnestic data were excluded from the evaluation process. Due to great inconsistency in the recording, classification was carried out based on morphologic findings only. Therefore, trauma and metabolic and toxic disturbances were not used as separate categories in the modified VITAMIN D scheme.

Cases with changes restricted to the pituitary gland, alterations of little to most likely no clinical significance, including mild hydrocephalus in brachycephalic dogs, and findings of presumably agonal origin were excluded from the evaluation. Similarly, cases only comprising meningitis or plexus choroiditis were not further assessed. Neoplasms and non-suppurative inflammation were further investigated by light microscopy and immunohistochemistry (see below). An evaluation regarding breeds and geographic distribution of disease patterns and occurrence of infectious agents was not performed due to missing data and inconsistent documentation.

### 2.2. Histopathology and Formalin-Fixed, Paraffin-Embedded Material

In recorded cases of non-suppurative encephalitis/myelitis or neoplasms, HE-stained sections of CNS tissue were used to reassess the diagnosis. In cases where no archived HE-stained sections were accessible, new sections were generated and stained using existing FFPE material whenever possible (previously described; [39]). Selected cases of CDV infections were additionally stained using Luxol fast blue cresyl violet to determine the occurrence of myelin loss. In cases of missing HE-stained sections and FFPE material for reevaluation, the documented diagnoses were accepted. The anatomical distribution of lesions could only be assessed approximately because the sampled CNS regions varied from case to case, and trimmed anatomical localization was not provided in most cases. Cases in which the previous neuropathologic diagnosis was not confirmed by reevaluation were either reclassified or, if no pathologic findings were found on the available slides, they were reclassified as cases with no significant microscopic lesions (NSML, Supplemental Figure S1).

### 2.3. Immunohistochemistry

Immunohistochemistry (IHC) was performed to confirm suspected specific viral infections. Table 2 summarizes the used primary antibodies, their targeted proteins, manufacturer, clonality, host species, pretreatment, and working dilution. Detection of viral antigens was performed using monoclonal antibodies targeting CDV nucleoprotein [40], parvovirus capsid antigen [41], and polyclonal antibodies targeting SHV-1-antigen [42], *Toxoplasma* (*T*.) *gondii*-antigen [43], with cross-reactivity to other apicomplexan parasites, and rabies virus nucleoprotein [44]. IHC staining was restricted to CNS tissue.

To distinguish between primary and secondary CNS neoplasms in cases without a definitive diagnosis based on the HE-stained section, IHC was performed. The applied monoclonal antibodies were used to identify oligodendrocytes using oligodendrocyte transcription factor 2 (Olig2) [45] and cyclic-nucleotide 3′-phosphodiesterase (CNPase) [46], cluster of differentiation 3 (CD3) [47] for T-lymphocytes, paired box protein 5 (PAX5) [48] for B-lymphocytes, CD 204 (CD204) [49] for histiocytic cells, and synaptophysin [50] for neuro-endocrine cells. Using polyclonal antibodies, astrocytes were identified with an antibody recognizing the glial fibrillary acidic protein (GFAP) [51], and histiocytes with one recognizing ionized calcium-binding adapter molecule 1 (IBA 1) [52]. For negative controls, Balb/c mouse ascitic fluid (diluted 1:1000 in PBS) or rabbit serum (diluted 1:3000 in PBS) were used instead of the primary antibody. A biotinylated goat anti-mouse antibody (Vector Laboratories Inc., Newark, CA, USA) or a biotinylated goat anti-rabbit antibody (Vector Laboratories Inc.), both diluted 1:200 in PBS, served as secondary antibodies. Sections were counterstained with Mayer’s hematoxylin (Carl Roth GmbH & Co. KG, Karlsruhe, Germany).

For evaluation, sections were exclusively qualitatively analyzed regardless of the antibody used and tissue examined. Positive controls included the cerebellum of a CDV-infected dog, brain stem and trigeminal ganglion of an SHV 1-infected dog, brain stem of a rabies-infected cow, small intestine of a dog infected with canine parvovirus as well as brain of a *T. gondii*-infected cat. A morphologically unaltered lymph node of a dog served as a positive control for IBA-1, PAX5, CD3, and CD204. Non-lesioned canine brain was used as control tissue for Olig2, CNPase, synaptophysin, and GFAP.

## 3. Results

### 3.1. Neuropathologic Findings in Dogs

**Figure 1:**
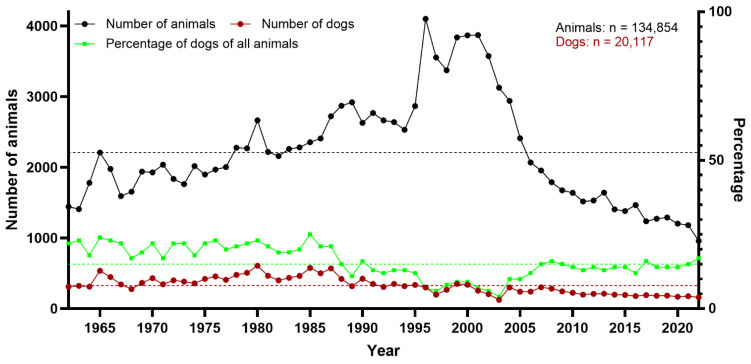
Total number of necropsied animals overall, and total number and percentage of dogs necropsied between 1962 and 2022 at the Department of Pathology, University of Veterinary Medicine Hannover, Hannover, Germany. Stippled lines represent the arithmetic means (black: all animals [*n* = 2211]; red: dogs [*n* = 329]; green: percentage of dogs of all animals [15%]).

During the 61-year study period (1962–2022), a total of 134,854 animals were submitted for necropsy to the Department of Pathology, University of Veterinary Medicine Hannover, including 20,117 dogs, various farm and companion animals, and a wide range of zoo animals and native wildlife species. This resulted in arithmetic means of about 2211 animals and 329 dogs per year. However, these numbers were not distributed equally over the years within the observation period (Figure 1). There was a peak of more than 3000 necropsied animals per year between 1996 and 2004 and a continuous decline in more recent years, with fewer than 1000 necropsies in 2022. The highest number of necropsy cases was recorded in 1996 (*n* = 4102), and the lowest in 2022 (*n* = 959). Between 1977 and 2006, the number of necropsied animals was consistently above 2000.

**Figure 2:**
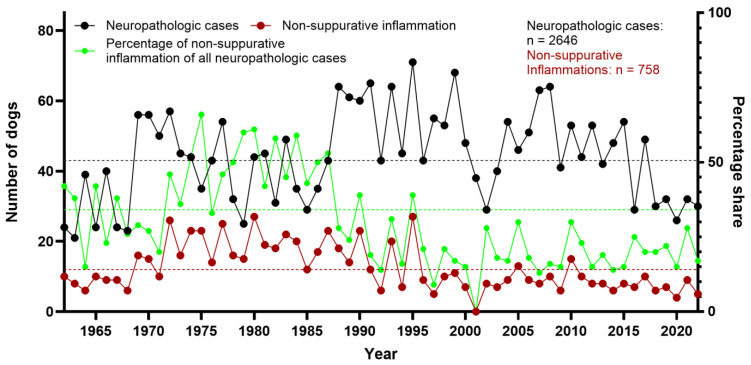
Total number of necropsied dogs, and total number and percentage of neuropathologic cases between 1962 and 2022 at the Department of Pathology, University of Veterinary Medicine Hannover, Hannover, Germany. Stippled lines represent the arithmetic means (black: all dogs [*n* = 329]; red: neuropathologic cases [*n* = 43]; green: percentage of neuropathologic cases of all dogs [13%]).

The mean percentage of necropsied dogs within the 61-year period was 15%. The highest number of dogs (*n* = 607) was counted in 1980, whereas the lowest number of dogs (*n* = 126) was recorded in 2003. From 1962 to 1987, the percentage of dogs among all necropsied animals was between 17% and 25%. Thereafter, it remained between 4% and 17%. Of all necropsied dogs in the studied time period, 13% (*n* = 2646) were assessed as neuropathologic cases. The highest peak was seen in 2003 (31% of all canine cases submitted for necropsy) and the lowest in 1965 (4%). While the absolute number of dogs showed a decline over the years, the number of neuropathologic cases appeared more constant, resulting in a relative increase in neuropathologic cases (Figure 2).

### 3.2. Categorization of Canine Neuropathologic Cases

**Figure 3:**
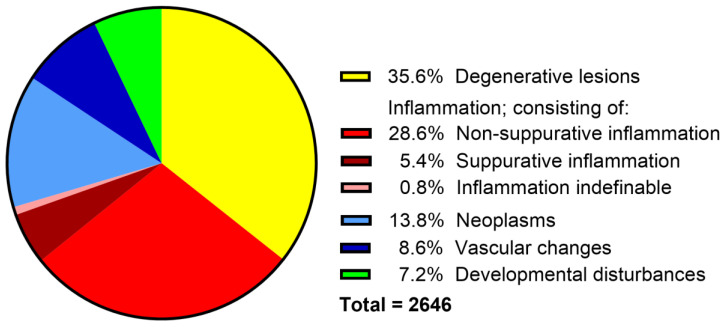
Classification and percentage of the different neuropathologic findings in dogs necropsied between 1962 and 2022 at the Department of Pathology, University of Veterinary Medicine Hannover, Hannover, Germany.

Degenerative lesions represented 35.6% (*n* = 943), non-suppurative inflammation 28.6% (*n* = 758), neoplasms 13.8% (*n* = 364), vascular disorders 8.6% (*n* = 227), congenital malformations 7.2% (*n* = 190), and suppurative encephalitis/myelitis 5.4% (*n* = 142) of all neuropathologic cases. In 0.8% (*n* = 22) of cases, the type of inflammation could not be determined due to insufficient information in archived documentation and lack of material for reevaluation (Figure 3).

### 3.3. Non-Suppurative Inflammation

**Figure 4:**
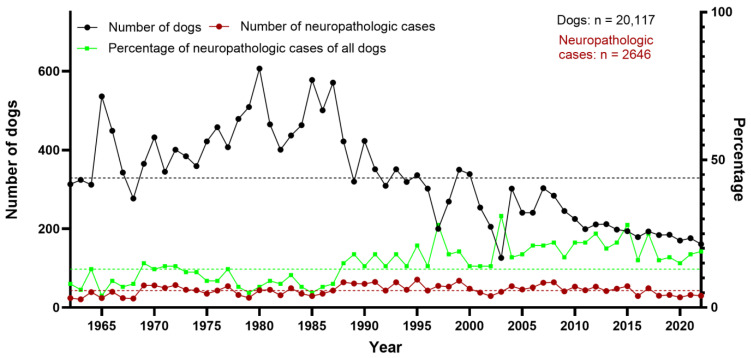
Total number of neuropathologic cases in dogs, and total number and percentage of cases of non-suppurative inflammation between 1962 and 2022 at the Department of Pathology, University of Veterinary Medicine Hannover, Hannover, Germany. Stippled lines represent the arithmetic means (black: all neuropathologic cases [*n* = 43]; red: non-suppurative inflammation [*n* = 13]; green: percentage of non-suppurative inflammation cases of all neuropathologic cases [30%]).

In 28.6% (*n* = 758) of dogs, non-suppurative inflammation was diagnosed. In the first half of the investigated time period (1962–1991), non-suppurative encephalitis/myelitis was the most common neuropathologic finding, accounting for 35.8% of all cases, with the highest percentage in 1975 (64%) (Figure 4). In the second half of the investigated time span (1992–2022), 19.6% of neuropathologic cases were diagnosed with non-suppurative inflammation. The year with the lowest number of cases was 2001, without a single case of non-suppurative encephalitis/myelitis, followed by 2022, with five cases (17% of dogs with neuropathologic diagnosis, Figure 4). Additionally, various causes of the observed non-suppurative encephalitis/myelitis were identified, the most commonly diagnosed being CDV and SHV.

### 3.4. Morbillivirus Canis Infection

**Figure 5:**
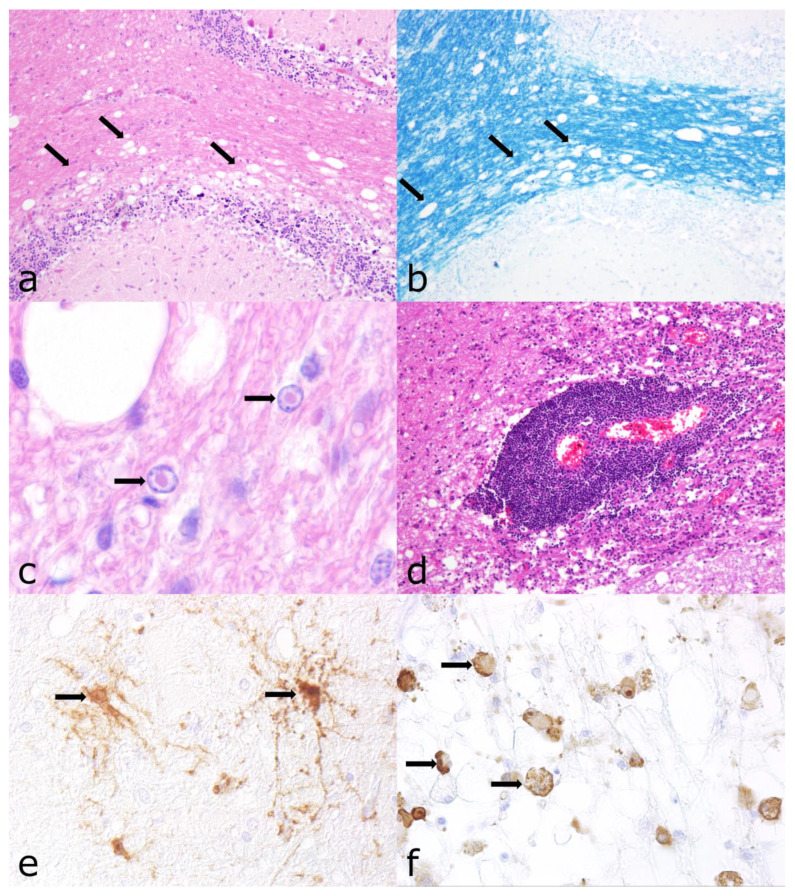
Characterization of cases with non-suppurative encephalomyelitis due to canine distemper virus (CDV) infection. (**a**) Cerebellum; multifocal, mild to moderate vacuolization of white matter (arrows) in early subacute CDV infection with minimal inflammation and lack of eosinophilia indicating myelin loss, hematoxylin and eosin (HE; ×100). (**b**) Multifocal mild demyelination (arrows), Luxol fast blue-cresyl violet (LFB; ×100). (**c**) Intranuclear, eosinophilic inclusion bodies (arrows) in cells resembling astrocytes surrounded by a clear halo, HE; ×1000. (**d**) Severe lympho-histiocytic perivascular infiltration in chronic CDV infection, HE; ×100. (**e**) CDV antigen found in cells resembling astrocytes (arrows), immunohistochemistry (IHC) for CDV antigen, Mayer’s hematoxylin counterstain; ×400. (**f**) CDV antigen was found in cells resembling macrophages (arrows), IHC for CDV antigen, with Mayer’s hematoxylin counterstain; ×400.

An infection with CDV was the cause of non-suppurative inflammation in 25% (*n* = 186) of cases. These cases were either suspicious for a CDV infection in the archived records or had lesions consistent with a CDV infection characterized by leukoencephalitis with and without demyelination or polioencephalitis. Demyelination was further investigated using Luxol fast blue cresyl violet staining (Figure 5). In addition, characteristic distemper findings included intranuclear and cytoplasmic, eosinophilic inclusion bodies. Only cases with lesions in the CNS were included in this study. Cases with CDV-induced lesions in extra-neural tissues without CNS involvement were excluded from the study.

**Figure 6:**
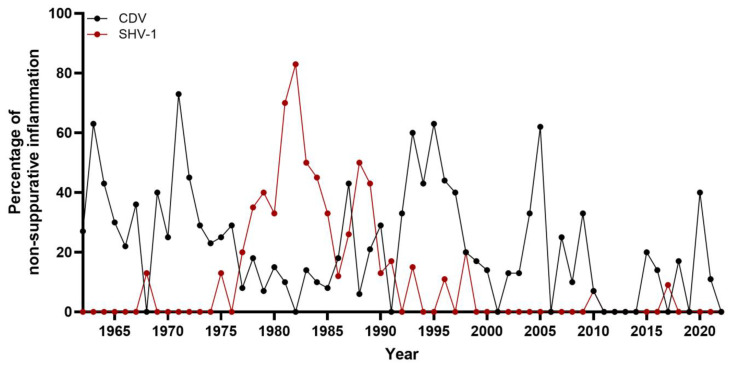
Percentage of cases with Morbillivirus canis (CDV) and Varciellovirus suidalpha (SHV-1) infections of all canine cases with non-suppurative encephalitis/myelitis in dogs necropsied between 1962 and 2022 at the Department of Pathology, University of Veterinary Medicine Hannover, Hannover, Germany.

While distemper has rarely been diagnosed in the last two decades (2000–2022), it was a rather common diagnosis in necropsied dogs in the years before. CDV was the most commonly found causative agent of non-suppurative encephalitis/myelitis between 1962 and 1969. During this time period, CDV-associated neuropathologic changes comprised 39% cases of non-suppurative inflammation. During the 1970s, distemper accounted for 28% of non-suppurative encephalitis/myelitis cases (Figure 6). In 1971, the highest percentage of CDV infections comprising 80% of all non-suppurative encephalitis/myelitis cases was documented. In the 1980s, the percentage dropped to 15%, rising again in the 1990s to 39%. In 1995, the highest absolute number of CDV CNS infections was reported, with 17 positive cases representing 63% of non-suppurative encephalitis/myelitis cases. Distemper was also detected in two thirds of neuropathologic cases in 2005 (*n* = 8). Since then, CDV infection has become a sporadic diagnosis, with zero to two cases per year (Figure 6).

In 86 cases, CDV antigen detection was performed previously and recorded in the archived documents. In 23 of these cases, immunofluorescence (IF) was performed, with the earliest IF testing for CDV dating back to 1976. It continued to be the method of choice for CDV diagnosis until the early 1990s. In the other 63 cases, IHC was applied, which replaced IF in the early 1990s as the most common test method. All formerly verified cases were accepted as positive cases without retesting. A further 54 cases with suspected CDV infection were investigated for this study using IHC. IHC resulted in immunolabeling of viral antigen characterized by a dark brown, granular cytoplasmic and/or intranuclear signal. Viral antigens were found mainly in astrocytes, but also in cortical neurons, neuronal and glial processes, endothelial cells as well as in microglia/macrophages (Figure 5).

Taken together, 140 of the 186 distemper cases tested positive for CDV, and the remaining 46 lacked suitable FFPE material to perform IHC. A total of 20 of these cases could be evaluated using HE-stained tissue; however, the other 26 lacked both HE-stained slides and FFPE material so the diagnosis of a CDV infection was based solely on the archived diagnosis.

Co-infections with other pathogens were documented twice with apicomplexan parasites. One case was confirmed by IHC for both CDV and Apicomplexa (1981). The second case with an apicomplexan co-infection was suspected (1993) but could not be substantiated due to the lack of FFPE material.

### 3.5. Varicellovirus Suidalpha1 Infection

The second most commonly detected cause of non-suppurative encephalitis/myelitis was SHV-1, the causative agent of Aujeszky’s disease. This pathogen was found in 16% (*n* = 119) of the cases.

**Figure 7:**
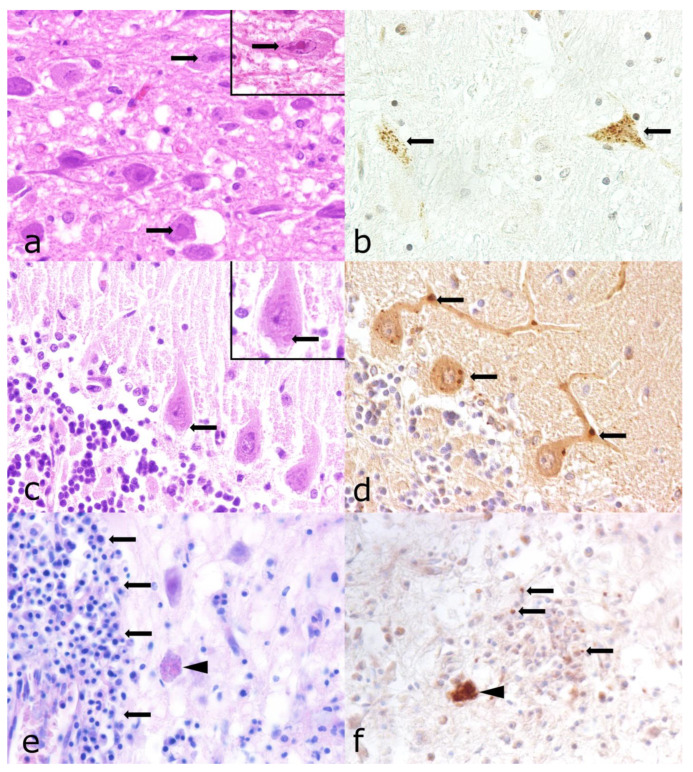
Histologic and immunohistochemical characterization of encephalitides caused by viral and apicomplexan agents. (**a**) Brain stem, numerous neurons with intranuclear inclusion bodies (arrows, inset) in a dog infected with Varicellovirus suidalpha1 (SHV-1), hematoxylin and eosin (HE; ×400). (**b**) SHV-1 antigen was found in neurons of the brain stem (arrows), immunohistochemistry (IHC) for SHV-1 antigen, with Mayer’s hematoxylin counterstain; ×400. (**c**) Cerebellum, eosinophilic, cytoplasmic inclusion body (Negri body) within Purkinje cells (arrow, inset) in a dog with rabies, HE; ×400. (**d**) Rabies antigen was found in Purkinje cells and their axons (arrows), IHC for rabies antigen, with Mayer’s hematoxylin counterstain; ×400. (**e**) Cerebrum, lymphoplasmacytic inflammation (arrows), and protozoal cyst (arrowhead), HE; ×400. (**f**) Extracellular tachyzoites positive for *Toxoplasma gondii* antigen (arrows), and protozoal cyst (arrowhead), IHC for *T. gondii*-antigen, with Mayer’s hematoxylin counterstain; ×400.

Selection and diagnostic criteria of the archived cases during the first screening included characteristic morphologic findings, including mild to moderate and occasional perivascular, lymphohistiocytic inflammation in the brain stem, inconstantly accompanied by the formation of glial nodules and/or a positive virus culture result for SHV-1 performed by the State Veterinary Investigation Office. Eosinophilic, intranuclear inclusion bodies were rare (Figure 7).

While the first case of SHV-1 in a dog dates back to 1968, the increased percentage of SHV-1 infected cases in the late 1970s to early 1990s was striking (Figure 6). During the 1980s, SHV-1 was detected in 45% of all cases with non-suppurative inflammation. The highest absolute numbers of cases were found in 1981 (*n* = 14) and 1982 (*n* = 15), corresponding to the highest percentage of affected dogs with non-suppurative encephalitis/myelitis, representing 74% and 83%, respectively. Between 1975 and 1993, 113 SHV-1 cases (95% of all cases observed between 1962 and 2022) were recorded. In the remaining time intervals, SHV-1 infections were diagnosed only sporadically (0 to 2 cases/year).

A total of 73.9% (*n* = 88) of 119 SHV-1 cases were previously tested positive by the State Veterinary Investigation Office. An additional 24 cases (20.2%) were confirmed using IHC in the scope of the present study. Immunohistochemical labeling of the SHV-1 antigen was characterized by a dark brown, granular cytoplasmic and intranuclear signal in neurons and, less frequently, in glial cells. The signal was not consistently associated with the inflammatory response (Figure 7).

The remaining 5.9% (*n* = 7) of cases were considered positive based on recorded histologic lesions and interpretation alone, as no tissue samples and slides were available, and these cases had not been tested previously. Co-infections with other infectious agents were not found.

### 3.6. Lyssavirus Rabies Infection

Infection with rabies virus was diagnosed in 1% (*n* = 8) of cases of non-suppurative inflammation within the study period, the last one being reported in 1985. Seven of these cases were confirmed using immunofluorescence by the State Veterinary Investigation Office, whereas the first case from 1964 was identified as rabies solely by histologic detection of Negri bodies. Three rabies cases, two from 1974 and one from 1980, were confirmed by IHC within the scope of this study. The others lacked HE-stained tissue slides as well as FFPE material to substantiate the original diagnosis. All three immunohistochemically confirmed rabies cases showed inflammatory alterations in the brain stem and to a lesser extent in other brain areas. These changes consisted of moderate perivascular cuffing and small glial nodes, historically termed Babes nodules, as well as small, eosinophilic cytoplasmic inclusion bodies in Purkinje cells and other neurons (Negri bodies) (Figure 7). A brown, granular immunohistochemical signal was found in the perikaryon of neurons in areas of inflammation, as well as in CNS compartments without lesions. Co-infections with other known infectious agents were not found.

### 3.7. Other Detected Pathogens

Several other infectious agents were recorded sporadically. CHV infection was diagnosed in 0.5% (*n* = 4) of cases of non-suppurative encephalitis, all of which had been confirmed by PCR prior to this study. Tick-borne encephalitis virus (TBEV) infection had been confirmed in two cases prior to this study, the former by IHC and in the latter by reverse-transcription PCR (0.26% of non-suppurative encephalitis cases). Concerning apicomplexan parasites (*n* = 13; 1.7% of non-suppurative encephalitis cases), nine had been identified as *Neospora (N.) caninum* and two as *T. gondii* by IHC prior to this study. In two additional cases, protozoan cysts were present within areas of inflammation, but FFPE material for IHC was not available anymore. All cases showed a granulomatous to necrotizing inflammation of either the brain and/or the spinal cord, and parasitic structures were documented in some cases. The immunohistological signal revealed extracellular tachyzoites and was limited to the areas of inflammation (Figure 7). Co-infection with CDV was shown via IHC in one case (1981) and was suspected in a second one (1993).

In two additional cases (0.3% of non-suppurative encephalitis cases), cerebrospinal nematode infection or migrating nematode larvae with associated multifocal eosinophilic inflammation were recorded. Furthermore, a single case of both protothecosis and cryptococcosis (each 0.1% of non-suppurative encephalitis/myelitis cases) was reported.

### 3.8. Exclusion of Parvovirus Infection

Six cases of non-suppurative inflammation and one case with necrotic changes in the CNS displayed lesions in the small intestine and lymphoid tissues that were strongly suggestive of a canine parvovirus infection. All cases had already been tested as immunohistologically positive for parvovirus in extraneural tissues. Parvovirus antigen was not detected immunohistologically in CNS tissue of any of these cases.

### 3.9. Non-Infectious, Non-Suppurative Encephalomyelitis of Unknown Origin

**Figure 8:**
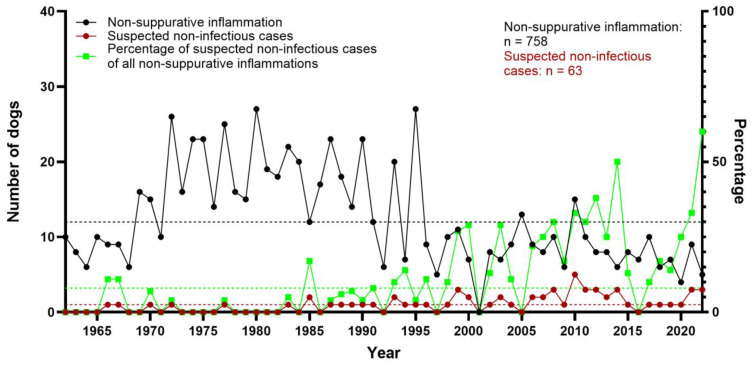
Total number of canine non-suppurative inflammation cases, and total number and percentage of suspected non-infectious cases between 1962 and 2022 at the Department of Pathology, University of Veterinary Medicine Hannover, Hannover, Germany. Stippled lines represent the arithmetic means (black: all non-suppurative inflammation cases [*n* = 13]; red: suspected non-infectious cases [*n* = 1]; green: percentage of non-infectious cases among all cases of non-suppurative inflammation [8%]).

Granulomatous and/or necrotizing inflammation of unknown origin (meningoencephalitis of unknown origin, MUO) was diagnosed in 8.3% (*n* = 63) of non-suppurative encephalitis/myelitis cases. These cases comprised granulomatous meningoencephalitis (GME; *n* = 49), necrotizing meningoencephalitis (NME; *n* = 7), and necrotizing leukoencephalitis (NLE; *n* = 6). In addition, one case displayed eosinophilic meningoencephalitis most likely of non-infectious origin due to lack of specific histologic findings and negative results for *T. gondii*, *N. caninum*, and CDV. Over the years, an increase in cases likely of non-infectious origin was noted, with only 11 cases seen before 1990 (Figure 8). Since 2000, 41 cases were documented. These likely non-infectious cases in different forms have been the most common diagnosis in non-suppurative encephalitis/myelitis during this time span.

### 3.10. Neoplasms

Neoplasms of the CNS were diagnosed in 13.8% (*n* = 364) of all canine neuropathologic cases. Primary tumors were further distinguished from secondary neoplasms, the latter originating from extraneural tissue and reaching the CNS either hematogenously or by local extension [53]. Tumors originating from tissues adjacent to the CNS, e.g., pituitary gland, were disregarded unless they infiltrated the CNS tissue. Tumor-associated lesions, e.g., compression-induced demyelination, were counted as degenerative changes if the neoplasm itself did not reach the CNS.

**Figure 9:**
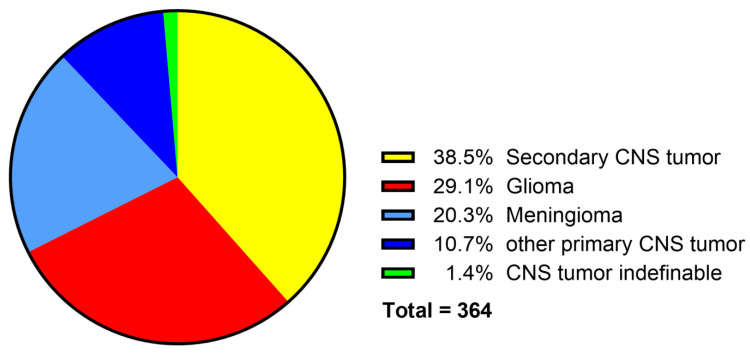
Classification of central nervous system (CNS) neoplasms in dogs necropsied between 1962 and 2022 at the Department of Pathology, University of Veterinary Medicine Hannover, Hannover, Germany.

Primary neoplasms of the CNS comprised 60.2% (*n* = 219) of all neoplasms, including 107 gliomas, 73 meningiomas, 13 choroid plexus papillomas, 8 primary histiocytic sarcomas, 6 neoplasms of embryonic origin, 5 primary lymphomas, 3 choroid plexus carcinomas, 2 thoracolumbar spinal tumors in young dogs, 1 suprasellar germ cell tumor, and 1 microgliomatosis (Figure 9). In 38.5% (*n* = 140) of cases, a secondary neoplasm of the CNS was present, either as locally invasive tumor or as metastasis. In 1.4% of the cases (*n* = 5), the tumor was not classifiable because the archived report was vague and no FFPE material for further investigation was available (Figure 9).

The number of neoplasms varied strongly from year to year. Nonetheless, an increase in both absolute numbers and percentage was seen from the late 1990s onward. In the second half of the investigated time period, 1992 to 2022, 77% (*n* = 281) of neoplasms were recorded, including 164 primary CNS neoplasms.

## 4. Discussion

The reevaluation of neuropathologic cases in dogs over a 61-year period revealed degenerative lesions and non-suppurative encephalomyelitis as the two most common alterations. Several infectious pathogens were detected, with CDV and SHV-1 being the most frequently diagnosed infectious agents.

To categorize the different neuropathologic findings, the VITAMIN D scheme was modified to classify neuropathologic cases without taking anamnestic or clinical data into consideration [6]. This was necessary to achieve a level comparability across 61 years of archived data.

Most neuropathologic diagnoses in the study population were categorized as degenerative alterations (35.6%). It was not differentiated whether a degenerative change represented a primary or secondary CNS alteration, for example, in metabolic diseases. These results are similar to a study from Berne, Switzerland (1989–2000), which reported degeneration in 38% of cases, and a former study from Hannover, Germany (1998–2003), which found degeneration accounting for 24.8% of cases; however, there are slight differences in the definitions of degeneration between the studies [27,28]. In particular, the inclusion of traumatic and metabolic and toxic lesions in the category needs to be taken into account in the present study, as it prevents distinct interpretation of these etiologies and the occurrence of primary degenerative lesions.

The second largest category were cases of non-suppurative inflammation (28.6%) of either the brain, spinal cord, or both. Compared to other studies, this percentage is relatively high. A former investigation from Hannover (1998 to 2003) found non-suppurative encephalitis/myelitis covering 21.8% of cases and representing the third largest group after degeneration and neoplasms [28]. An even smaller proportion is documented in the aforementioned study from Switzerland (1989 to 2000), which found non-suppurative inflammation as the second largest category, occurring in 14% of cases [27]. This can be explained by the higher percentage of non-suppurative inflammation in the first half of the investigated time span (36% in 1962–1991 vs. 20% in 1992–2022), as both other studies did not include data from years before 1989. One reason for this shift is the decline in CDV and SHV1 infections in the second time period, the two most frequently diagnosed causes of non-suppurative encephalitis and myelitis in the present study.

The most commonly detected infectious agent was CDV, found in 25% of non-suppurative inflammatory cases. This finding aligns with previous studies, which showed that CDV is among the most threatening virus in dogs and was one of the most common occurring diseases until into the 1990s [54,55,56]. Interestingly, CDV infections were found considerably less in the 1980s than in the 1970s or 1990s. Several factors might contribute to these differences. Several vaccinations, almost exclusively life vaccines, were available for dogs in the 1950s [26,57,58]. Already in the 1960s, reference was made to the decline in canine distemper cases, probably due to rising rates of vaccination in comparison to the higher number of cases in the 1950s [26,59]. The continuing incidence of distemper infections, albeit in smaller numbers, has been linked to a lack of compliance with the vaccination schedules, vaccines that do not offer complete protection, and inadequate immunization [60,61]. A rise in CDV cases was also described in studies from the 1990s and was associated with various factors, including the drop in vaccination rates and incomplete vaccination schedules, as well as potential vaccination failures [30,54,55,58]. Since 2005, CDV has become a sporadic diagnosis in reinvestigated cases only.

The second most frequent virus infection was SHV-1, detected in 16% of non-suppurative inflammatory cases. Almost all of these cases occurred between 1975 and 1993, with only sporadic cases before and after this period. The first detected case dates back to 1968 when SHV-1 infections were still rarely documented in domestic pigs in Germany [62]. In dogs, infected pork was almost the only route of infection before the virus had been eradicated in domestic pigs [38,63,64]. Therefore, the rise in cases in the mid-1970s and the decline in infection numbers in the 1990s are both closely linked to the number of infected pigs during this time [62]. The last case of SHV-1 infection in a domestic pig in Germany was reported in April 2000 according to the Office International des Epizooties (OIE); now known as World Organization for Animal Health (WOAH) [65]. Afterwards, the infection numbers in dogs dropped to almost zero [64]. The two cases, which were diagnosed after 2000, were hunting dogs with direct contact to wild boars, which are still carriers of the virus [38,66].

Several other infectious agents were found in smaller numbers. Eight cases of rabies were detected in the archived records, three of which could be confirmed by IHC. The last rabies case in a domestic dog in this study dates back to 1985. Infected wildlife represented the main source of infections in Germany before the country was declared free of sylvatic rabies in 2007 [33,35,37]. Even though Germany has been considered rabies-free in all species but bats since 2007, a potential risk of rabies infections remains due to imports of dogs from countries which are not rabies-free [67,68].

Interestingly, only a few cases of CHV were found. It must be noted that this virus was not explicitly searched for in this study and was only included if CHV was already detected in extraneural tissues or suspected according to the archived records, which might underestimate the true prevalence of this virus. In a study from the University of Georgia, Athens, GA, USA, CHV represented the most frequent viral cause of non-suppurative encephalitis cases between 2008 and 2019 [29]. This might be associated with the geographic distribution as well as differences in vaccination strategies, study population, or occurrence of virus variants. Other studies from Europe document no cases of CHV in non-suppurative inflammation cases of the CNS [10,16,27,28].

The other sporadically found infectious agents include apicomplexan parasites as well as TBEV, cryptococcus, and prototheca. Due to the limited and partly non comparable data over the decades, these rare diseases cannot be assessed in terms of their prevalence. Other studies also documented these agents rarely or not at all [10,16,27,28,29].

Inflammation of most likely non-infectious origin, including GME, NLE, NME, and one case of eosinophilic inflammation, accounted for 8.3% (*n* = 63) of cases. Interestingly, almost two-thirds of these cases (*n* = 41) were found after 2000. In the older cases, the term “reticulosis” was frequently used to describe these cases [69,70]. This term, which was not exclusively used to describe inflammatory alterations but might also refer to neoplastic lesions, was replaced by the term GME [71,72]. An increased research interest in these lesions, both clinically and pathologically, at the University of Veterinary Medicine Hannover after 2000 certainly influenced the numbers, which has to be considered, especially when comparing the present study to other retrospective investigations [17,73]. However, non-infectious cases were reported as the most common form of non-suppurative inflammation of the CNS in dogs by several studies after 2000, indicating an actual rise in these cases for unknown reasons [10,17,73,74,75,76,77].

Taking all investigated infectious agents together, 334 (44.1%) of non-suppurative encephalitis/myelitis cases were etiologically resolved. A total of 63 (8.3%) cases showed the histopathologic pattern of GME, NLE, NME, or eosinophilic encephalitis of likely non-infectious origin [6,30,69,73,78]. The remaining 361 (47.6%) cases of non-suppurative inflammation remained etiologically undetermined. The number of cases labeled as undetermined varies greatly between studies, and comparisons should be drawn carefully due to different methodologies and classification criteria, e.g., definition and diagnosis of non-infectious origin or whether the diagnosis is based on pathological or clinical findings [16,29]. A former study from Germany (1998–2003) documented more etiologically undetermined cases (75%) [28], and a study from Switzerland (1988–1993), only 15% [10].

Neoplasms of the CNS accounted for 13.8% of neuropathologic cases and were divided into 219 primary CNS and 140 secondary neoplasms, respectively, with 5 cases that could not be further classified [53]. A total of 77% (*n* = 281) of the cases occurred in the second half of the investigated period after 1992. In other studies, the percentage of neoplasms in all neuropathologic cases varied between 9% and 22% [27,28]. The occurrence of primary and secondary tumors differed widely between various canine studies, from an almost equal number to a large overrepresentation of primary neoplasms [6,28,79,80]. Likely, this is mainly influenced by geographic distribution of certain breeds, their popularity in the investigated year, and their predisposition towards CNS neoplasms, as well as by varying definitions of primary and secondary [79,80,81]. The increase in cases in the second half of the investigated period is mainly linked to enhanced research interests in this specific topic at the University of Veterinary Medicine Hannover, which must be considered when comparing the present data to other retrospective studies [82,83].

The number of examined animals varied significantly over the years. The most prominent finding is the peak between the mid-1990s and the early 2000s, followed by a continuous decline in cases submitted for necropsy. The high numbers of examined animals in the mid-1990s is mainly explained by a high number of necropsied pigs in this period. The decline in overall numbers of necropsies in recent years, below the numbers of the 1970s and even the 1960s, affects both farm and companion animals and has different reasons depending on the species. In dogs, this decline started in the early 1990s. Our own experience and personal communication with clinicians suggest that owners more often prefer to bury or cremate their animals rather than giving consent for pathological examination. This variation in animal numbers over the years might influence the significance of disease trends seen in the present study.

Interestingly, this overall decline in submitted necropsy cases is not as pronounced as for dogs with neuropathologic changes. This is largely associated with unique research projects on neurologic diseases in dogs at the University of Veterinary Medicine Hannover. Further factors, such as a shift in the popularity of certain breeds that might show a predisposition of CNS diseases, cannot be excluded but lie beyond the scope of this study [10,15,84].

The high value of long-term stored FFPE material to provide information about the emergence and disappearance of diseases is impressively demonstrated in this study. These samples are often the only available source of material to investigate past outbreaks of diseases, occurrence and disappearance of certain entities, and epidemiologic changes with respect to infectious and non-infectious manifestations. This study again demonstrates their value in detecting known viral infections and further investigating so far unrecognized pathogens, even if information regarding the pre-fixation time and fixation time in formalin, as well as long-term storage conditions, is unavailable [85,86,87,88,89,90,91]. Nonetheless, an adverse influence of the overall quality of the FFPE material, especially regarding the prolonged storage time and missing information about the handling of the probes prior to fixation and/or embedding on IHC investigation in this study and other analyses cannot be ruled out.

Despite the points mentioned above, a few others need to be considered when comparing data over six decades that affect the interpretation and comparability of the results of the present study. The decision not to include any clinical and anamnestic data was necessary to achieve comparability over the decades but compromises the understanding of contextual factors between clinic and pathology, which would be of high value. Unfortunately, anamnestic reports were scarce or missing in many cases and were therefore not useful for this analysis. Secondly, due to missing information, breed and geographic data were excluded from evaluation, which could yield valuable information for pathologists and clinicians. Another factor of interest is that the distribution of lesions within the brain could not be assessed due to missing data and inconsistent sampling protocols. It was also decided to include cases which lacked both HE-stained slides and FFPE material, based on their archived diagnosis in the final evaluation process. This likely led to a slight inaccuracy in the case numbers because some lesions may have been under- or misdiagnosed.

This retrospective 61-year study on dogs with neuropathologic findings reflects the course of various CNS diseases, especially of non-suppurative inflammation, and thereby provides a reliable database for further investigations. The 47.6% etiologic unresolved cases of non-suppurative encephalitis demonstrate the complexity of these patterns of these diseases, the challenges in investigating such cases, and the need for further studies with advanced technologies using molecular analyses.

## Figures and Tables

**Table 1:** Comparison of VITAMIN D scheme 1 and the modified classification used for neuropathological assessment in the present study.

	Category	Type of Lesion According to Vandevelde et al. [6]	Modified for the Current Neuropathological Classification
V	Vascular diseases	Vasculitis, hemorrhage, infarction	Same categories
I	Inflammatory diseases	Parenchymal invasion of blood-derived leucocytes around blood vessels, infiltrating into the parenchyma, proliferation of endogenous microglia	Same categories
T	Trauma	Mechanical disruption of tissue compounded by traumatic injury to blood vessels	Not used; included in the category vascular and/or degenerative diseases
A	Anomalies	Abnormal development of the CNS in utero	Same categories
M	Metabolic/toxic diseases	Loss of architecture of nervous tissue caused by deficiencies and toxins	Not used; categorized as degenerative disease, included in category D
I	Idiopathic changes	Abnormal functional neurological signs without morphologically detectable changes in the CNS tissue	Not used, not applicable for this neuropathological study
N	Neoplasia	Destructive invasion or compression by tumor cell proliferation by primary or secondary CNS tumors	Destructive invasion or cell proliferation by primary or secondary CNS tumors; compression of tumors outside the CNS were classified as degenerative disease
D	Degenerative diseases	Progressive degeneration of various specific cell types in the nervous system in a bilaterally symmetrical and restricted anatomical localization, mostly connected to genetic defects	Encephalo- and myelomalacia; neuronopathies and axonopathies; spongy encephalomyelopathies; degenerations caused by vertrebral disc herniations; cerebellar abiotrophy; disruption of tissue

CNS; central nervous system; VITAMIN D, vascular diseases, inflammatory diseases, trauma, anomalies, metabolic/toxic diseases, idiopathic changes, neoplasia, degenerative diseases.

**Table 2:** Antibodies used for immunohistochemistry.

Specificity	Target	Dilution	Pretreatment	Clonality, Host Species	Source
CDV nucleoprotein (D110)	CDV	1:1000	Citrate buffer (0.01 mol/L), MW	Monoclonal, mouse	Kindly provided by University of Bern, Bern, Switzerland
SHV-1-antigen	SHV1	1:100	TRIS-EDTA buffer (0.01 mol/L; 0.001 mol/L), MW	Polyclonal, rabbit	Abcam, Cambridge, UK
*T. gondii*, cross-reaction with other apicomplexa	Apicomplexian parasites	1:75	None	Polyclonal, rabbit	Quartett, Potsdam, Germany
Rabies virus nucleoprotein	Rabies virus	1:500	Proteinase K	Polyclonal, rabbit	Flarebio Biotech LLC, Washington, DC, USA
CPV-antigen, cross-reaction with FPV; PPV and MPV	CPV	1:500	Proteinase K	Monoclonal, mouse	Custom Monoclonal International, Sacramento, CA, USA
PAX5	B-lymphocytes	1:500	Citrate buffer (0.01 mol/L), MW	Monoclonal, mouse	BD, Franklin Lakes, NJ, USA
CD3	T-lymphocytes	1:200	Citrate buffer (0.01 mol/L), MW	Monoclonal, rat	Bio Rad, Hercules, CA, USA
CD204	Histiocytic cells	1:500	Citrate buffer (0.01 mol/L), MW	Monoclonal, mouse	Abnova, Taipei City, Taiwan
IBA 1	Histiocytic cells	1:1000	Citrate buffer (0.01 mol/L), MW	Polyclonal, rabbit	Thermo Electron LED GmbH, Langenselbold, Germany
Olig 2	Oligodendrocytes	1:100	Citrate buffer (0.01 mol/L), MW	Monoclonal, rabbit	Abcam, Cambridge, UK
GFAP	Astrocytes	1:1000	None	Polyclonal, rabbit	Dako, Santa Clara, CA, USA
Synaptophysin	Neuro-endocrine cells	1:500	Citrate buffer (0.01 mol/L), MW	Monoclonal, mouse	Dako, Santa Clara, CA, USA
CPNase	Oligodendrocytes	1:500	Citrate buffer (0.01 mol/L), MW	Monoclonal, mouse	Chemicon International, Temecula, CA, USA

CD3, cluster of differentiation 3; CD204, cluster of differentiation 204; CDV, canine distemper virus; CPNase, 2′,3′-Cyclic-nucleotide 3′-phosphodiesterase; CPV, canine parvovirus; EDTA, ethylenediaminetetraacetic acid; FITC, fluorescein isothiocyanate; FPV, feline parvovirus; GFAP, glial fibrillary acidic protein; IBA1, ionized calcium-binding adapter molecule 1; MPV, mink parvovirus; MW, microwave (800 W); *N. caninum*, *Neospora caninum*; Olig 2, oligodendrocyte transcription factor 2; PAX5, paired box protein 5; PPV, porcine parvovirus; SHV-1, Varicellovirus suidalpha1; *T. gondii*, *Toxoplasma gondii*; TRIS, Tris(hydroxymethyl)aminomethane.

## Data Availability

The data supporting this study’s findings are available from the corresponding author upon reasonable request.

## Supplementary Materials

The following supporting information can be downloaded at https://www.mdpi.com/article/10.3390/vetsci12090869/s1, Figure S1: Visualization of classification of canine neuropathologic cases in the scope of this study. Neuro-pathologic cases were sorted according to a modified VITAMIN D1 scheme, with exclusion of the categories trauma, metabolic/toxic diseases, and idiopathic diseases to obtain a comparable database over the entire time span. CNS, central nervous system; FFPE, formalin-fixed, paraffin-embedded; HE, hematoxylin and eosin; IHC, immunohistochemistry; NSML, no significant morphologic lesion; VITAMIN D, vascular diseases, inflammatory diseases, trauma, anomalies, metabolic/toxic diseases, idiopathic changes, neoplasia, degenerative diseases. (1) By Vandevelde, M., Higgins, R. J. and Oevermann, A. Veterinary Neuropathology Essentials of Theory and Practice. (2012).
