# Supplementary figures and images for: Retrospective Analysis of Central Nervous System Diseases in Dogs, with Special Focus on Non-Suppurative Encephalomyelitis (1962–2022)
Source: Vet Sci. 2025 Sep 8;12(9):869. doi: 10.3390/vetsci12090869 (PMC12474455; doi:10.3390/vetsci12090869)

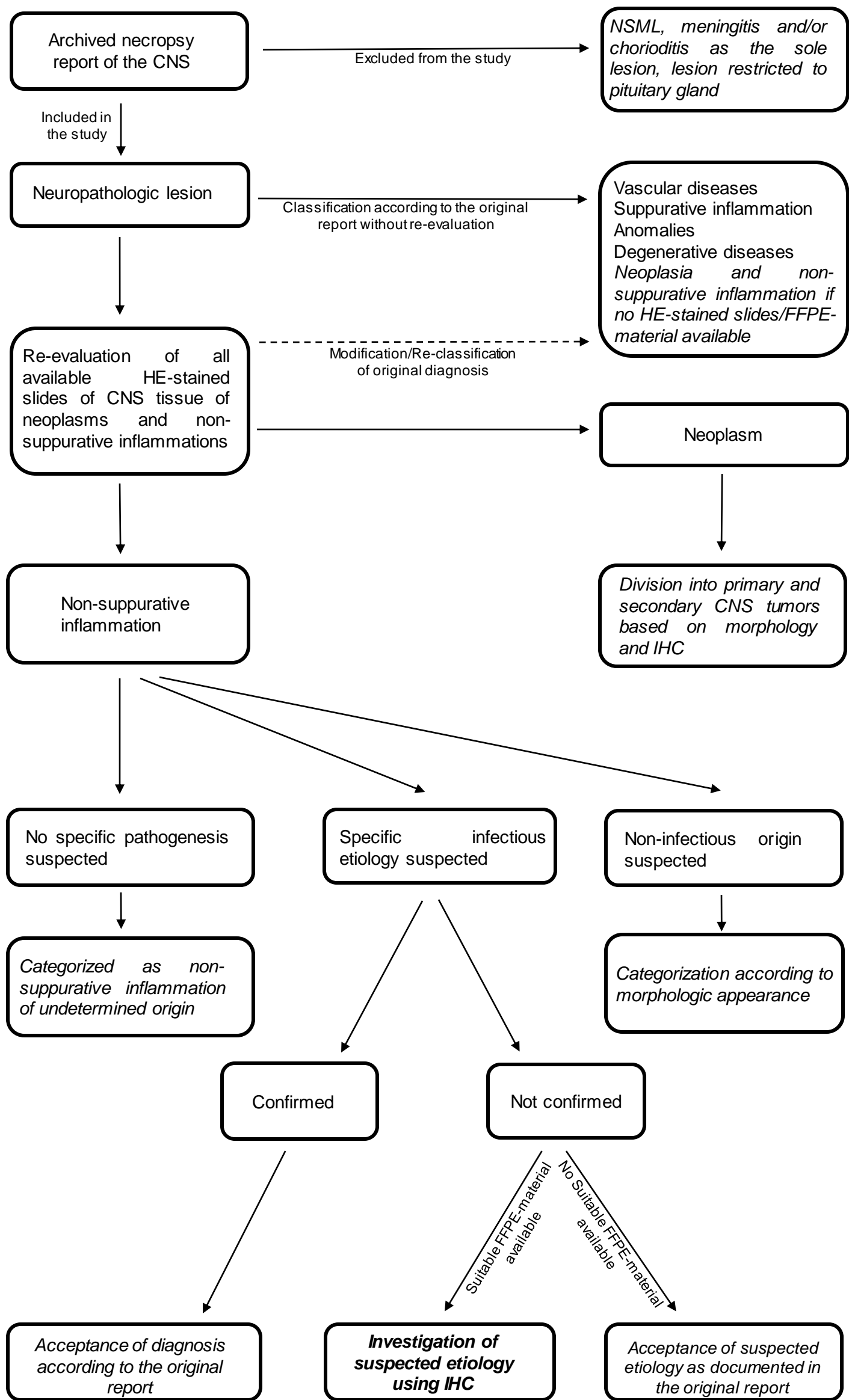

Supplement: Supplementary file 1 [file vetsci-12-00869-s001.zip › vetsci-3829810-supplementary.pdf]
